# Mortality patterns in municipalities of a mining region before the Brumadinho dam failure, state of Minas Gerais, Brazil

**DOI:** 10.1590/1980-549720230010.supl.1

**Published:** 2023-04-21

**Authors:** Deborah Carvalho Malta, Gabriela Maciel dos Reis, Guilherme Augusto Veloso, Laís Santos de Magalhães Cardoso, Zulmira Maria de Araújo Hartz, Matthew Cunningham, Mohsen Naghavi

**Affiliations:** I Universidade Federal de Minas Gerais, Department of Maternal and Child Nursing and Public Health – Belo Horizonte (MG), Brazil.; II Universidade Federal de Minas Gerais, School of Nursing, Graduate Program in Nursing – Belo Horizonte (MG), Brazil.; III Universidade Federal Fluminense, Institute of Mathematics and Statistics, Department of Statistics — Niterói (RJ), Brazil.; IV Universidade Nova de Lisboa, Institute of Hygiene and Tropical Medicine — Lisbon, Portugal.; V University of Washington, Institute for Health Metrics and Evaluation – Seattle (WA), United States of America.; VI University of Washington, Institute for Health Metrics and Evaluation, Department of Global Health – Seattle (WA), United States of America.

**Keywords:** Dam failure, Mining, Ecological studies, Mortality, External causes, Diagnosis of health situation

## Abstract

**Objective:**

To describe the patterns of overall mortality and mortality from external causes and the temporal evolution in the municipalities of the Paraopeba River Basin, before the socio-environmental disaster of the Brumadinho dam and, additionally, to investigate the correlation between mortality and socioeconomic deprivation in these municipalities.

**Methods:**

Global Burden of Disease Study mortality estimates for 26 municipalities in the state of Minas Gerais, Brazil, were analyzed. Rates of overall mortality and mortality from external causes were estimated in the triennia (T) T1 (2000 to 2002), T2 (2009 to 2011), and T3 (2016 to 2018). Pearson’s correlation coefficient measured the association between mortality rates and socioeconomic deprivation, according to the Brazilian Deprivation Index (IBP).

**Results:**

There was a decrease in overall mortality in the Paraopeba River Basin from 717.7/100 thousand to 572.6/100 thousand inhabitants, and in most municipalities between T1-T3. Mortality from external causes increased from 73.3/100 thousand to 82.1/100 thousand, and it was higher in these municipalities compared with the mean for Brazil and Minas Gerais. Deaths from suicide and interpersonal violence increased from 29.6/100 thousand to 43.2/100 thousand in most of the 26 municipalities. Death rates due to unintentional injuries decreased during the period, and those due to transport injuries, increased. There was a positive correlation between socioeconomic deprivation and the percent change in mortality rates.

**Conclusion:**

Despite the strong presence of mining activity in the region, such did not reflect in the improvement of the sanitary situation. Death rates due to external causes increased in the period, associated with inequalities, which must be considered in the planning for the recovery of the disaster areas.

## INTRODUCTION

The failure of Dam I to store tailings from the Córrego do Feijão Mine, in the municipality of Brumadinho, state of Minas Gerais (MG) – Brazil, in 2019, operated by the mining company Vale S.A., caused one of the most serious disasters in the world related to mining dams. Considered the largest work-related accident in Brazil, it fatally victimized 272 people, including 250 direct and outsourced employees of Vale S.A.^1,2^. The disaster caused the release of at least 12 million cubic meters of tailings on the ground and on the Paraopeba River, which reached more than 160 km in length^3^. These tailings resulted in environmental damage to vegetation and fauna, as well as the release of heavy metals into the water, such as manganese, aluminum, iron, and arsenic, restricting its use and the supply of water to the metropolitan region of Belo Horizonte (MG)^3,4^. In this course, in addition to Brumadinho, 25 other municipalities in the state^5^ were considered to be affected, corresponding to a population of about 1.1 million inhabitants.

Overall, disasters exceed the capacity of the affected community or society to face the situation with their own resources. That can increase the losses and damage to the environment and health of the very place of the event occurrence and its surroundings^6^. There are several resulting consequences, including material and economic losses as well as diseases, injuries, and deaths also at a time subsequent to the disaster^6^. Such circumstances require urgent decisions with the purpose of ceasing or reducing the environmental risks arising from contamination of the soil and watercourses and exposures to them and, consequently, of mitigating the damage to the ecosystem and health of populations, which may arise in the short-, medium-, and long-term^7^.

The scientific literature points to these changes in the morbidity and mortality patterns of populations affected by disasters, with an increase in the prevalence of chronic diseases and aggravation of previously-contracted diseases^7,8–15^, mental health impairment, increased consumption of alcoholic beverages and other drugs, and violence in the affected communities^16–20^. The occurrence of external causes is related to the process of loss of family members and loved ones and to the sudden rupture in social, economic, and also identity processes^21^. In order to dimension the impacts of a disaster, it is imperative to investigate the previous health conditions, vulnerabilities, and social and environmental contexts in which the affected population lived. Social inequalities and social vulnerability are assumed to result in worse health indicators in the region, prior to the disaster. We believe that knowing the mortality patterns and its temporal evolution before the disaster will allow us to outline the health status and identify temporal trends whose applicability — and relevance — is the establishment of a baseline for future assessments of the disaster impact on the health of affected populations.

The present study aims at describing the patterns of overall mortality and mortality from external causes and the temporal evolution in the municipalities of the Paraopeba River Basin, before the socio-environmental disaster of the Brumadinho dam and, additionally, to investigate the correlation between mortality and socioeconomic deprivation, according to the Brazilian Deprivation Index (*Índice Brasileiro de Privação –* IBP), in these municipalities.

## METHODS

### Study design and unit of analysis

This is an epidemiological study of the ecological type, descriptive and analytical, which investigated the overall mortality and mortality from external causes in 26 municipalities of the Paraopeba River Basin, state of Minas Gerais (MG) (Supplementary Material – Figure A), between 2000 and 2018, that is, in years prior to the failure of the mining tailings dam of Córrego do Feijão Mine.

### Data source

The database of deaths per municipalities used was elaborated in 2021 by researchers from the Institute for Health Metrics and Evaluation (IHME) of the University of Washington (USA), in the context of the Global Burden of Diseases (GBD) Study, on demand from the GBD Brazil Network.

The GBD Study uses national data collected from vital registration systems to estimate mortality and, with regard to Brazil, the data source is the Mortality Information System (*Sistema de Informações sobre Mortalidade* – SIM) of the Brazilian Ministry of Health^22^. Aiming to improve the quality of information, the IHME applies algorithms to correct the underreporting of deaths and to redistribute garbage codes (GC) to underlying causes of death, according to methods previously described in scientific publications^23,24^. Deaths classified as garbage mask the true underlying causes of death and correspond to codes of the International Classification of Diseases (ICD) that are: unspecified; intermediate or immediate causes of death; or impossible causes of death^23^.

The population estimates of the Brazilian Ministry of Health were used as denominators in the calculation of mortality rates and are available from the website of the Department of Informatics of the Brazilian Unified Health System — Datasus^25^.

### Indicators

Rates of overall mortality and mortality from external causes were estimated, expressed per 100 thousand inhabitants, for each of the 26 municipalities of the Paraopeba River Basin and for all these municipalities combined. For comparison purposes, mortality rates for all municipalities in the state of Minas Gerais and Brazil were also estimated. In this study, the external causes comprised three major groups of causes used by the GBD Study, at level 2 (two) of disaggregation^23^: a) self-harm and interpersonal violence; b) unintentional injuries; and c) transport injuries. To reduce random fluctuations, the rates were estimated by triennia: T1 (2000/2001/2002), T2 (2009/2010/2011), and T3 (2016/2017/2018). The numerator comprised the mean number of deaths and the denominator the mean population of each triennium. Rates were standardized for age by the direct method, using the standard population of the GBD study^23^.

The IBP was used as a measure of social inequality. The index was developed by researchers from the Center for Data and Knowledge Integration for Health (*Centro de Integração de Dados e Conhecimentos para Saúde* – Cidacs/Fiocruz Bahia) and the University of Glasgow. This is a socioeconomic deprivation index developed at the national level, estimated according to census tracts, and based on indicators from the 2010 demographic census related to *per capita* income, literacy, and housing conditions such as water, sanitation, and garbage collection^26^.

The indicators were summed up based on the estimation of the z-score: the “z,” for a variable “x,” was calculated using the formula z=(x−μ)/sd, where the mean “μ” and the standard deviation “sd” for each census tract indicator were weighted according to the population^26^. The final z-score value of each census tract was given by the simple sum of the z-score of the following indicators: income, level of education, and household conditions^21^. The index was also estimated at the municipal level using the same z-score method, enabling to classify each municipality with a score and to group them into deprivation quintiles, on a scale ranging from the lowest deprivation (-1.73) to the highest (+2.71). In the case of the 26 municipalities of the basin, they were classified between -1.38 (2^nd^ deprivation quintile) and -0.25 (4^th^ deprivation quintile) (Supplementary Material – Table A).

The IBP database by municipalities is available from the website of Cidacs/Fiocruz Bahia^27^.

### Data presentation and analysis

Municipal mortality rates were presented in tables and choropleth maps. Percent changes in mortality rates among the triennia were presented in heat maps, comparing the relative differences between the first and second triennia, the second and the third, and the first and third triennia. In the heat map, the values of the percent changes of the rates between triennia are presented in a two-by-two comparison (T1-T2, T2-T3, and T1-T3). These values are highlighted according to a color scale that represents the largest decreases (blue scale) and the largest increases (red scale) of the rates.

The correlation analysis considered the relationship between the percent change of mortality rates between T1 and T3, and the IBP. Pearson’s correlation coefficient was calculated, and statistically significant correlations were those whose p-value was less than or equal to 5%. The Dancey and Reidy^28^ classification was used to define the magnitude of the correlation: values lower than 0.30 are considered weak; between 0.40 and 0.60, moderate; and greater than 0.70, strong. Data presentation and analysis was performed in the statistical R software.

### Ethical aspects

This study integrates a broader project approved by the Research Ethics Committee of Universidade Federal de Minas Gerais under Opinion No. 3.258.076. The study used records of secondary data of non-nominal basis, which do not allow for the identification of individuals, in accordance with Decree No. 7,724, May 16, 2012^29^, and Resolution No. 510 of April 7, 2016^30^.

## RESULTS

**Figure 1:**
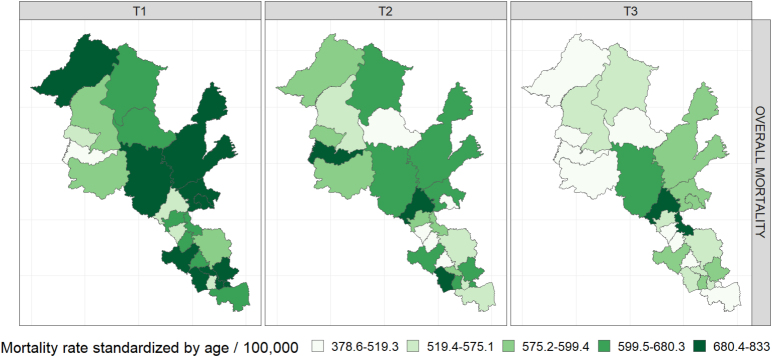
Municipal rates of overall mortality, standardized by age, per 100 thousand inhabitants, in the triennia T1 (2000/2001/2002), T2 (2009/2010/2011), and T3 (2016/2017/2018), Paraopeba River Basin, state of Minas Gerais, Brazil.

The mean overall mortality rates for all 26 municipalities of the Paraopeba River Basin decreased from 717.7/100 thousand inhab. (T1) to 572.6/100 thousand inhab. (T3). The same occurred for Brazil, from 771.8/100 thousand inhab. (T1) to 622.6/100 thousand inhab. (T3), and for the state of Minas Gerais (Table 1). There was a decrease in overall mortality in the period in almost all municipalities, except Fortuna de Minas, Papagaios, Paineiras, and Igarapé, whose trend was upward (Figure 1 and Supplementary Material – Table B).

**Table 1:** Rates of overall mortality and mortality from external causes, standardized by age, per 100 thousand inhabitants, in the triennia T1 (2000/2001/2002), T2 (2009/2010/2011), and T3 (2016/2017/2018), Brazil, state of Minas Gerais, and municipalities of the Paraopeba River Basin.

Location	Overall mortality			External causes			Self-harm and interpersonal violence			Unintentional Injuries			Transport injuries		
	T1	T2	T3	T1	T2	T3	T1	T2	T3	T1	T2	T3	T1	T2	T3
Brazil	771.8	669.6	622.6	84.9	79.3	75.1	37.6	34.8	36.8	21.8	20.2	18.5	25.5	24.3	19.7
Minas Gerais	698.8	606.0	553.1	62.2	70.9	64.4	22.4	29.9	29.2	18.7	17.4	16.2	21.1	23.6	18.9
Paraopeba River Basin*	717.7	625.2	572.6	73.3	91.2	82.1	29.6	45.5	43.2	20.1	18.1	17.5	23.7	27.7	21.4

* Corresponding to the set of 26 municipalities of the Paraopeba River Basin.

**Figure 2:**
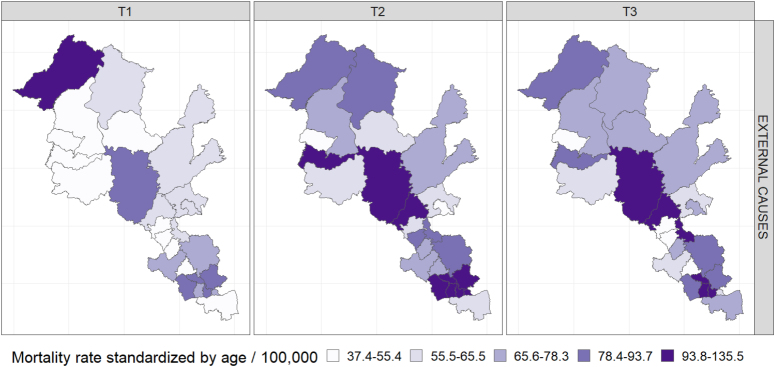
Municipal rates of mortality from external causes, standardized by age, per 100 thousand inhabitants, in the triennia T1 (2000/2001/2002), T2 (2009/2010/2011), and T3 (2016/2017/2018), Paraopeba River Basin, state of Minas Gerais, Brazil.

Regarding mortality from the set of external causes, the inverse occurred, with an increase in mortality rates in the Paraopeba Basin of 73.3/100 thousand inhab. (T1) to 91.2/100 thousand inhab. (T2), and 82.1/100 thousand inhab. (T3). The rates for Brazil decreased from 84.9/100 thousand inhab. (T1) to 75.1/100 thousand inhab. (T3). In Minas Gerais, it remained stable from 62.2/100 thousand inhab. (T1) to 64.4/ 100 thousand inhab. (T3) (Table 1). Figure2 shows higher rates in the municipalities of the Paraopeba Basin in the last two triennia compared with the first one. Among the municipalities of the Paraopeba Basin, the rates increased over the three triennia in most municipalities and some with very high rates, such as: Papagaios, Fortuna de Minas, and Pompéu in T3 (135.5 per 100 thousand inhab., 117.1 per 100 thousand inhab., and 114.6 per 100 thousand inhab., respectively) (Supplementary Material – Table B).

**Figure 3:**
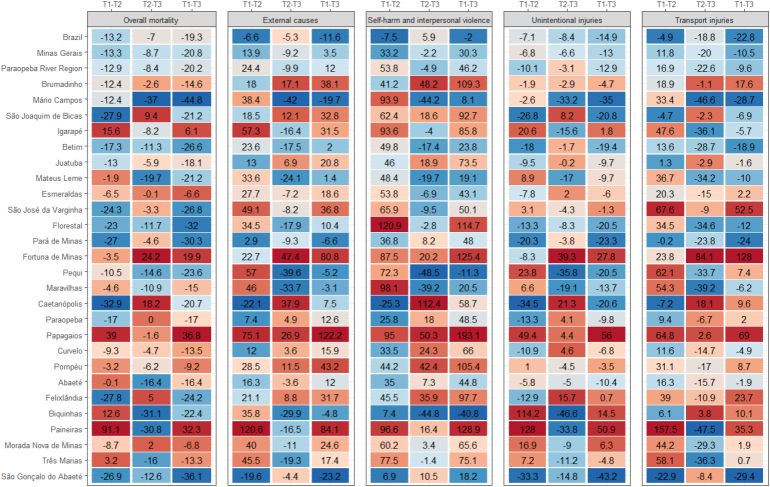
Percent change in rates of overall mortality, total and disaggregated mortality from external causes, between the triennia T1 (2000/2001/2002), T2 (2009/2010/2011), and T3 (2016/2017/2018), in Brazil, state of Minas Gerais, and municipalities of the Paraopeba River Basin.

Figure 3 shows the values of percent changes in mortality rates among the triennia. Regarding the set of external causes, we observed an increase of +24.4% in the Paraopeba Basin and, conversely, a decrease of -6.6% in Brazil, and an increase of +13.9% in MG. Between T1-T3, there was an increase of +12.0% in the Paraopeba Basin and +3.5% in MG and a decrease of -11.6% in Brazil. When analyzing the municipalities separately, there was an increase in rates among the triennia, especially T1-T2 in almost all municipalities, except Caetanópolis and São Gonçalo do Abaeté. The largest increases occurred in Paineiras (+120.6%), Papagaios (+75.1%), and Igarapé (+57.3%). In T1-T3, there was a slight decrease in a larger number of municipalities (Figure 3).

By evaluating external causes in a disaggregative manner, focusing on the set of 26 municipalities of the Paraopeba Basin, self-harm and interpersonal violence accounted for the higher burden of mortality, followed by transport injuries and, finally, unintentional injuries (Table 1). In the third triennium, the mortality rate due to self-harm and interpersonal violence was twice the value of the mortality rate due to transport injuries (43.2 vs. 21.4 per 100 thousand inhab.) and 2.5 times the mean mortality rate due to unintentional injuries (43.2 vs. 17.5 per 100 thousand inhab.) (Table 1). Concerning mortality from suicide and interpersonal violence, the Paraopeba Basin rates were higher than those for Minas Gerais and Brazil in the second and third triennia (Table 1). In the Paraopeba Basin, there was an increase of over 50% from the first to the second triennium and of 46.2% from the first to the third triennium, while in Brazil, there was stability in the percent change between T1-T2 and T1-T3 and, in Minas Gerais, there was an increase of over 30% from the first to the second triennium (Figure 3). Throughout the analyzed period, there was a significant increase in rates in most municipalities (Supplementary Material – Figure B, Table B; Figure 3).

Mortality rates due to unintentional injuries were similar for Brazil, Minas Gerais, and the Paraopeba Basin (Table 1). In Supplementary Material – Figure C, we can observe a darkening of the map from T1 to T2 and a lightening from T2 to T3, indicating, over the three periods, an increase in rates followed by a decrease. Rates decreased between T1 and T3 in most municipalities (n=14) and were stable in eight other municipalities (Supplementary Material – Table B; Figure 3).

As for mortality from transport injuries, the highest rates in T3 occurred in the Paraopeba Basin (21.4 per 100 thousand inhab.), compared with the mean rates for Brazil (19.7 per 100 thousand inhab.) and Minas Gerais (18.9 per 100 thousand inhab.) (Table 1). In Supplementary Material –Figure D, we can observe that rates increased in the municipalities especially between T1-T2, slightly decreasing in the last triennium. When comparing T1-T3, there was a decrease in rates due to transport injuries in 12 municipalities and an increase above 10% in eight municipalities (Figure 3).

**Figure 4:**
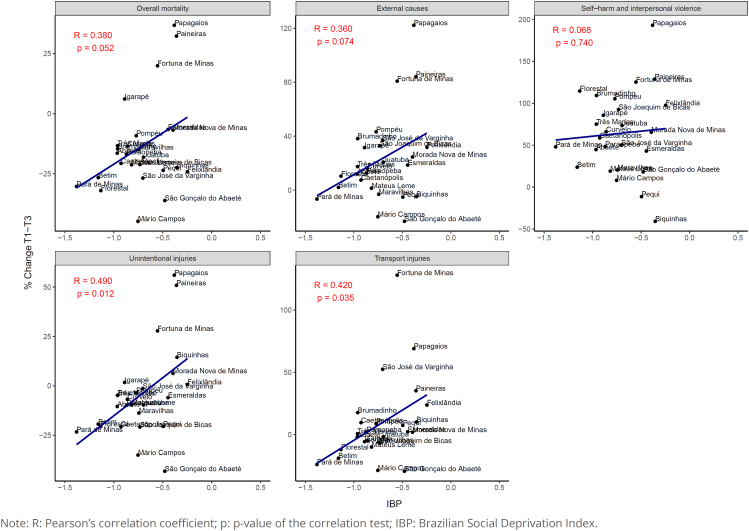
Scatter plot and correlation analysis between the percent change of rates of overall mortality and mortality from external causes in the triennia T1 (2000/2001/2002) and T3 (2016/2017/2018) and the Brazilian Deprivation Index of each municipality of the Paraopeba River Basin, state of Minas Gerais, Brazil.

Note: R: Pearson’s correlation coefficient; p: p-value of the correlation test; IBP: Brazilian Social Deprivation Index.

In the correlation analysis, we identified a positive, moderate, and statistically significant correlation between: percent change in mortality rates due to unintentional injuries and IBP (R=0.49; p=0.012); and percent change in mortality rates due to transport injuries and IBP (R=0.42; p=0.035) (Figure 4).

## DISCUSSION

We identified high rates of overall mortality and mortality from external causes in the municipalities of the Paraopeba River Basin, higher than the mean for Brazil and Minas Gerais in the period prior to the environmental disaster of the mining company Vale S.A. in Brumadinho. Among the external causes, deaths from self-harm and interpersonal violence were the highest and increased over the triennia in the 26 municipalities. Unintentional injuries decreased during the period, and transport injuries rates increased. The latter tended to increase as socioeconomic deprivation increased, according to IBP measures.

The disaster that occurred in 2019 in the municipality of Brumadinho demonstrates the negligence with environmental, social, health, and well-being issues of the affected population and does not comply with the country’s commitment to global pacts such as the Sendai Framework and the 2030 Agenda for Sustainable Development. The Sendai Framework for Disaster Risk Reduction aims to achieve, by 2030, the substantial reduction of disaster risk and losses in lives, with specific actions focused on good governance^31^. Target 3.9 of the Sustainable Development Goals (SDG) aims at substantially reducing the number of deaths and illnesses from hazardous chemicals and air, water, and soil pollution and contamination^32^.

A disaster has numerous consequences and repercussions for the affected people and communities. Likewise, and paradoxically, it also represents the possibility of demonstrating the historical and social conditions behind its origin and can serve to foster discussions for changing such conditions^33^. Before this disaster, communities already experienced vulnerabilities and lack of structures and public policies; the high mortality rates from violence reported in this study suggest this scenario. Furthermore, to understand the mortality patterns of a population, social processes should be considered^34^.

Based on the IBP, the municipalities with the highest socioeconomic deprivation presented higher rates of overall mortality and mortality from external causes in the pre-disaster period. The most violent municipalities of the basin can be divided into two groups: those closest to the metropolitan region of Belo Horizonte (Betim, Juatuba, Esmeraldas, and São Joaquim de Bicas) and municipalities that are located in the lower Paraopeba (Papagaios, Pompéu, and Paineiras). The second group is characterized by its strong relationship with agriculture and mining^3,35^.

Deaths from external causes in the Paraopeba Basin region increased in the period prior to the disaster, especially due to self-harm, interpersonal violence, and transport injuries. We highlight the increase in violence between the first and third triennia, much higher than the increase in rates in Minas Gerais and Brazil. These findings may reflect the contradiction already identified in other studies on economic development and mining. If, on the one hand, large mining projects generated economic growth, on the other hand, they did not foster greater well-being of the population in general^36^. It is noteworthy that mining activity is one of the main economic activities of the country, accounting for about 4.08% of the gross domestic product (GDP) in 2018. However, along with its economic impact, it presents significant socio-environmental externalities, environmental degradation, and socio-environmental disasters^37^.

A study conducted in the state of Pará (Brazil), on six municipalities where large mining companies are headquartered, identified that, despite the significant economic growth driven by the extractive industry, there was no change in the structural form of poverty conditions in the municipalities. On the contrary, there were setbacks from the point of view of social inequalities, with worsening of the Gini index between 1990 and 2010. In other words, mining activities increased the concentration of income and social inequalities^36^. Another study also highlights the low investment in the state’s regulatory capacity on mining activity, and the State is unable to adequately monitor and regulate this important economic activity^37^. Thus, these studies can help to understand the results in the Paraopeba Basin region. Despite the strong presence of mining activity in recent decades in the region and the increased rate of extraction, there was no improvement in its health situation, with structural problems, social inequalities, and local vulnerabilities persisting, which must be overcome.

Injuries and deaths from external causes result in social, health, and economic burden for individuals, families, the society, and the government, in addition to consisting in important public health issues in Brazil^38^. Brazil stands out for being one of the most violent countries in the world: the second with the most firearm deaths^39^ and one of the five countries with the highest rates of road traffic deaths. According to Minayo^40^, structural violence is institutionalized in society, economic, cultural, political, and family systems; it profoundly influences socialization practices and results in suffering and death.

Socioeconomic factors are directly related to violence; this also results from social inequalities and it mainly affects places of misery and poverty^41^. Therefore, to overcome this situation, it is necessary to strengthen health surveillance, foster intersectoral, interdisciplinary, and multiprofessional articulation and the organization of the civil and community society^42^, promote research to identify policies and strategies for preventing violence as well as support the monitoring and evaluation of the effectiveness of actions^40,43^.

The study has strengths, such as the use of GBD mortality data adjusted for underreporting and redistribution of garbage codes, which represents an advance in terms of improving the quality of mortality information. However, this is an ecological study, which has limits in its capacity of analysis and causality attribution. In addition, these data refer to small municipalities, and the rates may present fluctuations resulting from the small numbers in the numerator, which we sought to minimize by triennia aggregation. External causes were disaggregated at level 2 (two) of the GBD Study, which are more aggregated and include interpersonal violence and self-harm, although the highest fraction of death rates is due to physical aggression (data not shown). Another limitation concerns the IBP, whose indicators were calculated based on 2010 variables because there are no updated data from the demographic census. Therefore, we should consider the implication of the gap of these data for the classification of deprivation in the municipalities, which may result in bias in the vulnerability diagnosis. Finally, it is worth mentioning that the Pearson’s correlation coefficient only quantifies the degree of the linear relationship between two variables and that high correlations do not imply a cause-and-effect relationship.

All in all, despite the strong presence of mining activity in the Paraopeba Basin region, situations of social inequalities persist, and there was no improvement in the health situation in the period prior to the disaster. External causes have increased and are associated with social inequalities, which are accentuated in the region. These data should be considered in prevention measures to be implemented and can guide the allocation of public resources and other investments in the region, as compensation for the environmental damage caused. These results can inform stakeholders and support actions aimed at affected and more vulnerable population groups.
