# Supplementary material for: Mortality patterns in municipalities of a mining region before the Brumadinho dam failure, state of Minas Gerais, Brazil
Source: Rev Bras Epidemiol. 2023 Apr 21;26(Suppl 1):e230010. doi: 10.1590/1980-549720230010.supl.1 (PMC10176738; doi:10.1590/1980-549720230010.supl.1)
Supplement: Supplementary file 1 [file 1980-5497-rbepid-26-suppl1-e230010-suppl1.pdf]

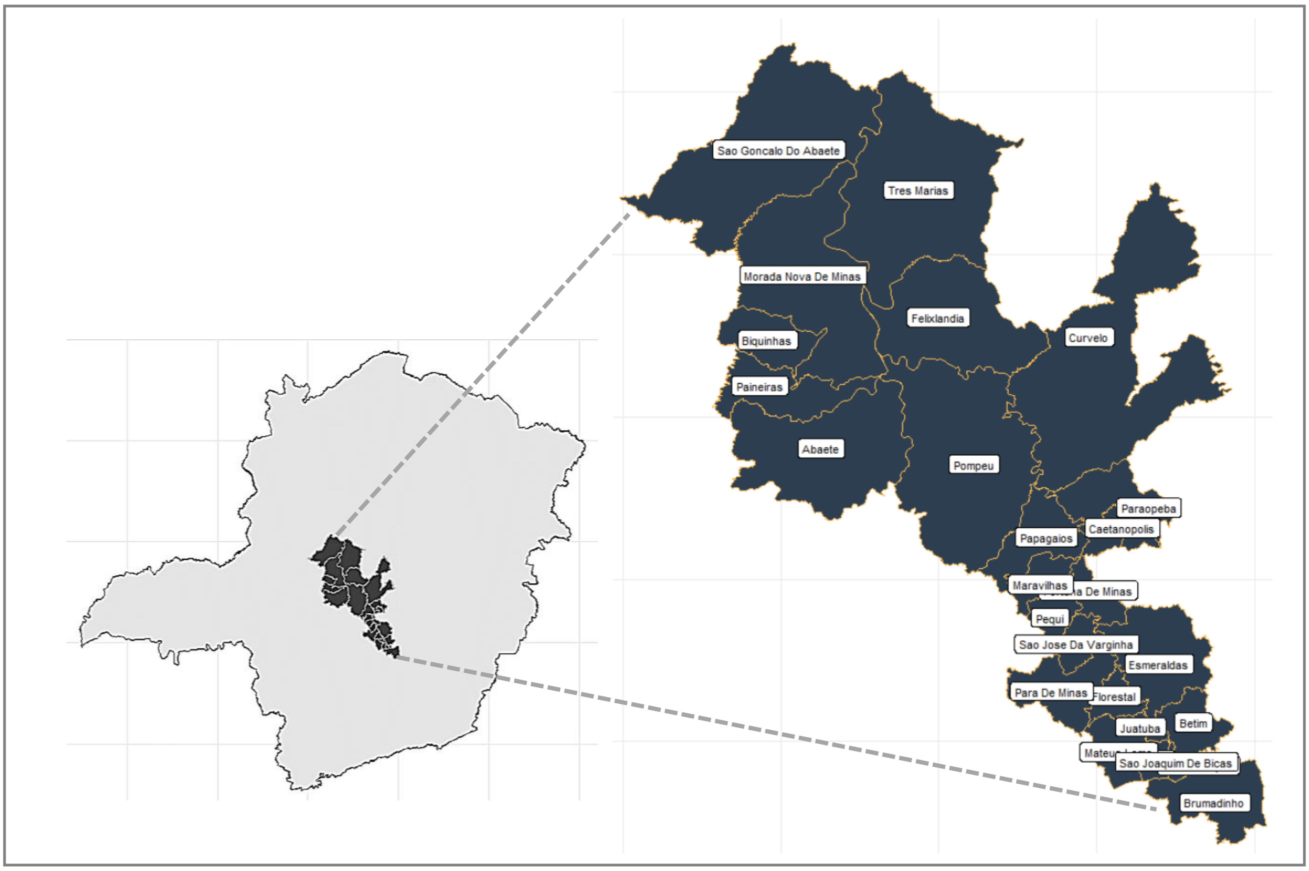

Figura A - Localização e identificação dos 26 municípios da Bacia Hidrográfica do Rio Paraopeba no estado de Minas Gerais

Tabela A – Valor e respectivo quintil do Índice Brasileiro de Privação (IBP) dos municípios que integram a Bacia Hidrográfica do Rio Paraopeba, Minas Gerais

Município	Valor do IBP	Quintil de Privação
Pará de Minas	-1,38	2
Betim	-1,16	2
Florestal	-1,13	2
Abaeté	-0,96	3
Três Marias	-0,96	3
Brumadinho	-0,96	3
Caetanópolis	-0,93	3
Igarapé	-0,89	3
Paraopeba	-0,87	3
Curvelo	-0,86	3
Mateus Leme	-0,82	4
Pompéu	-0,77	4
Mário Campos	-0,75	4
Maravilhas	-0,74	4
São Joaquim de Bicas	-0,73	4
São José da Varginha	-0,70	4
Juatuba	-0,70	4
Fortuna de Minas	-0,55	4
Pequi	-0,50	4
São Gonçalo do Abaeté	-0,48	4
Esmeraldas	-0,45	4
Morada Nova de Minas	-0,39	4
Papagaios	-0,38	4
Paineiras	-0,36	4
Biquinhas	-0,35	4
Felixlândia	-0,25	4

Tabela B - Taxas de mortalidade geral e por causas externas, padronizadas por idade, por 100 mil habitantes, nos triênios T1 (2000/2001/2002), T2 (2009/2010/2011) e T3 (2016/2017/2018), municípios da Bacia Hidrográfica do Rio Paraopeba.

Local	Mortalidade geral			Causas externas			Suicídio e violência interpessoal			Acidentes não intencionais			Acidentes de transporte		
	T1	T2	T3	T1	T2	T3	T1	T2	T3	T1	T2	T3	T1	T2	T3
Brumadinho	606,6	531,6	517,8	53,3	62,9	73,6	17	24	35,5	20,7	20,3	19,7	15,7	18,6	18,4
Mário Campos	752,9	659,6	415,6	71	98,2	56,9	21,9	42,4	23,7	26,9	26,2	17,5	22,2	29,6	15,8
São Joaquim de Bicas	725	522,6	571,6	86,9	103	115,5	38,1	61,9	73,4	24,7	18,1	19,5	24,2	23	22,5
Igarapé	552,2	638,2	585,9	71,6	112,6	94,2	27,4	53	50,9	21	25,4	21,4	23,2	34,2	21,9
Betim	813,4	673	596,7	83,7	103,4	85,3	41,1	61,6	50,9	20,7	17	16,7	21,8	24,8	17,7
Juatuba	680,5	592,3	557,1	87	98,3	105,1	28,4	41,4	49,2	23,1	20,9	20,9	35,5	36	35
Mateus Leme	713	699,3	561,5	83	110,9	84,2	32,3	47,9	38,5	22,7	24,7	20,5	28	38,3	25,2
Esmeraldas	582,1	544,6	543,9	71,1	90,8	84,3	32,4	49,8	46,3	19,9	18,3	18,7	18,8	22,7	19,3
São José da Varginha	669,4	506,6	489,8	52,5	78,3	71,9	10,2	16,8	15,2	14,8	15,3	14,6	27,6	46,2	42
Florestal	668,4	514,7	454,4	49,9	67,1	55	10	22,1	21,5	18,1	15,7	14,4	21,7	29,2	19,1
Pará de Minas	833	608,5	580,6	66,7	68,6	62,2	15,9	21,7	23,5	18,9	15,1	14,5	31,8	31,8	24,2
Fortuna de Minas	599,5	578,8	719	64,8	79,5	117,1	14	26,3	31,6	30,2	27,7	38,6	20,6	25,5	47
Pequi	557,7	499,1	426,1	53,7	84,3	50,9	18,2	31,4	16,1	12	14,9	9,6	23,5	38	25,2
Maravilhas	625,8	597,2	532,1	38,6	56,3	37,4	8,5	16,9	10,3	14,5	15,5	12,5	15,5	23,9	14,5
Caetanópolis	755,1	506,5	598,7	64,3	50,1	69,1	13,3	9,9	21,1	26,2	17,2	20,8	24,8	23	27,1
Paraopeba	721,9	599,5	599,4	57,6	61,8	64,8	17,7	22,3	26,3	17,9	15,5	16,1	22	24,1	22,4
Papagaios	548,4	762,4	750,1	61	106,8	135,5	27,5	53,7	80,7	13,3	19,9	20,8	20,1	33,1	34
Curvelo	682,4	619,2	590,4	63,7	71,3	73,8	19,2	25,6	31,8	17,6	15,7	16,4	26,9	30	25,6
Pompéu	703	680,3	638,3	80	102,8	114,6	31,1	44,9	64	20,5	20,7	19,8	28,3	37,1	30,8
Abaeté	593,8	593,5	496,2	54	62,8	60,5	19,1	25,8	27,7	16,3	15,4	14,6	18,6	21,6	18,2
Felixlândia	649,7	469,2	492,6	50	60,5	65,8	11,2	16,3	22,1	18,6	16,2	18,8	20,2	28	25
Paineiras	378,6	723,5	500,8	42,7	94,2	78,6	20,3	40	46,6	11,5	26,1	17,3	10,9	28	14,7
Biquinhas	521,7	587,2	404,7	40	54,3	38,1	12,6	13,6	7,5	10,8	23,2	12,4	16,5	17,5	18,2
Morada Nova de Minas	588,3	537,4	548	54,5	76,3	67,9	18,1	28,9	29,9	19	22,2	20,2	17,5	25,2	17,8
Três Marias	634,9	655,3	550,2	64	93,1	75,2	16	28,4	28	22	23,6	21	26	41,1	26,2
São Gonçalo do Abaeté	788,3	576,1	503,5	107	86	82,2	25,5	27,3	30,2	39,8	26,6	22,6	41,7	32,1	29,5

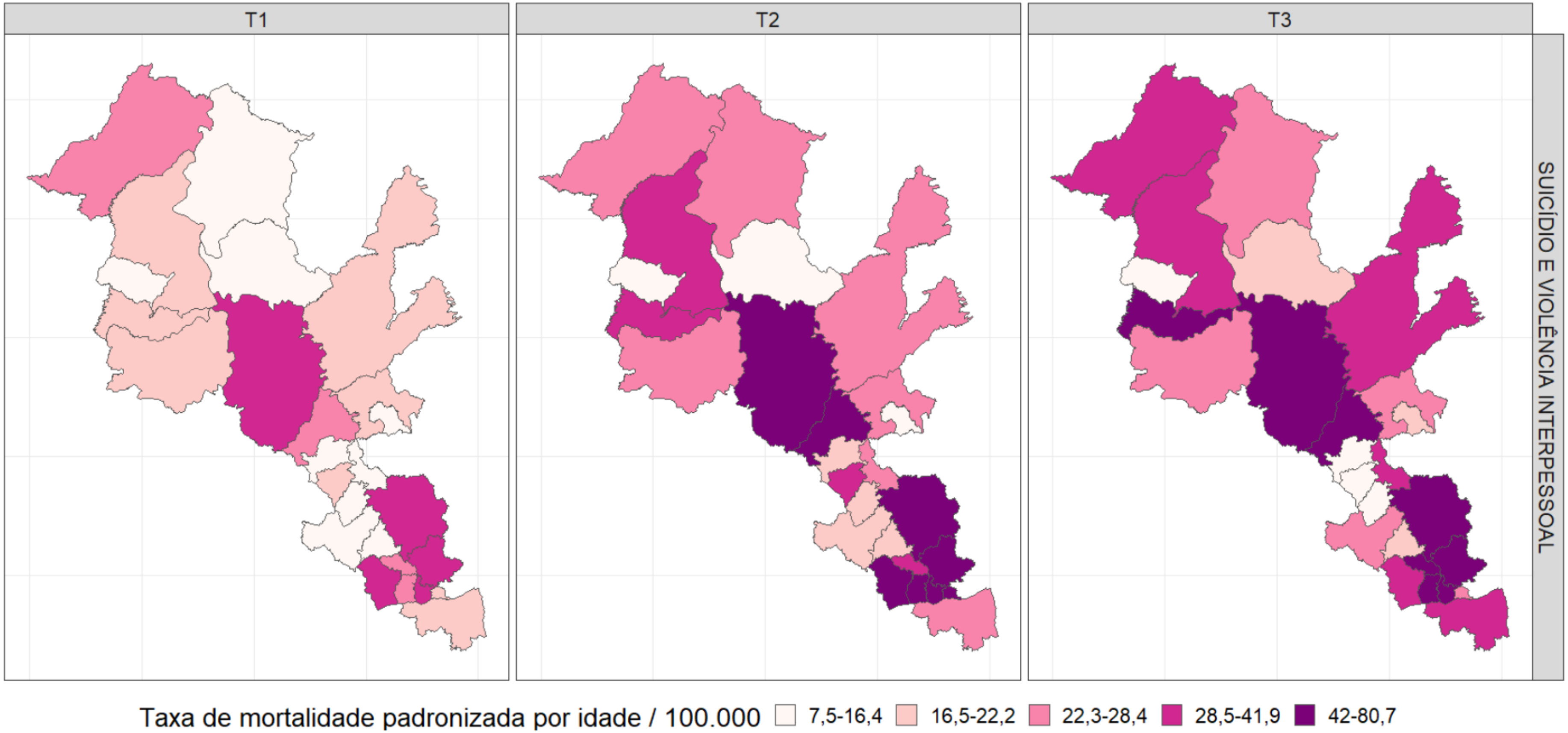

Figura A - Taxas municipais de mortalidade por suicídio e violência interpessoal, padronizadas por idade, por 100 mil habitantes, T1 (2000/2001/2002), T2 (2009/2010/2011) e T3 (2016/2017/2018), Bacia Hidrográfica do Rio Paraopeba, Minas Gerais

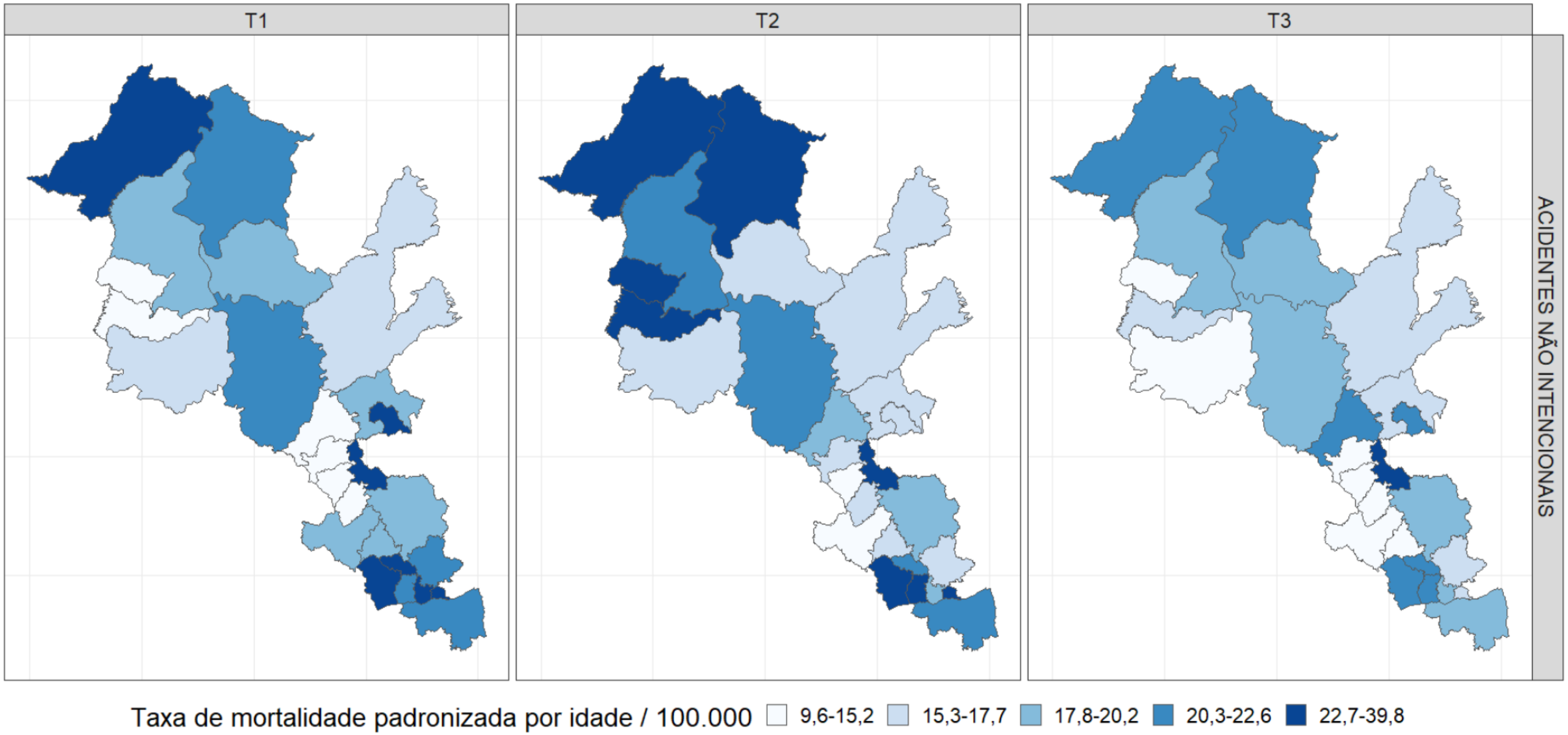

Figura B - Taxas municipais de mortalidade por acidentes não intencionais, padronizadas por idade, por 100 mil habitantes, T1 (2000/2001/2002), T2 (2009/2010/2011) e T3 (2016/2017/2018), Bacia Hidrográfica do Rio Paraopeba, Minas Gerais

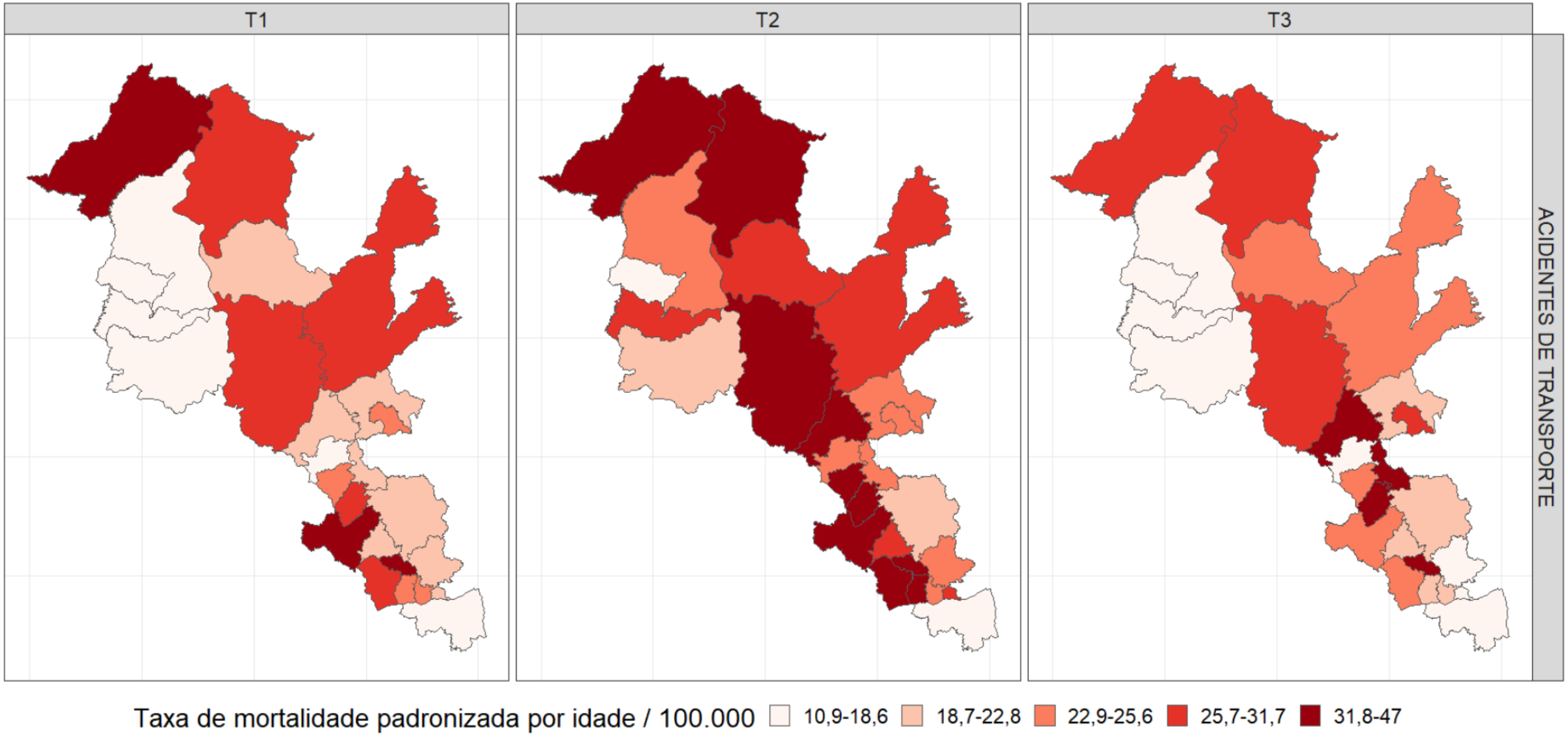

Figura C - Taxas municipais de mortalidade por acidentes de transporte terrestre, padronizadas por idade, por 100 mil habitantes, T1 (2000/2001/2002), T2 (2009/2010/2011) e T3 (2016/2017/2018), Bacia Hidrográfica do Rio Paraopeba, Minas Gerais
