# Supplementary material for: Mortality patterns in municipalities of a mining region before the Brumadinho dam failure, state of Minas Gerais, Brazil
Source: Rev Bras Epidemiol. 2023 Apr 21;26(Suppl 1):e230010. doi: 10.1590/1980-549720230010.supl.1 (PMC10176738; doi:10.1590/1980-549720230010.supl.1)
Supplement: Translation

# Translation: Padrões de mortalidade em municípios de uma região mineradora antes do rompimento da barragem de Brumadinho, Minas Gerais, Brasil

**DOI:** 10.1590/1980-549720230010.supl.1.1

**Authors:** Deborah Carvalho Malta, Gabriela Maciel dos Reis, Guilherme Augusto Veloso, Laís Santos de Magalhães Cardoso, Zulmira Maria de Araújo Hartz, Matthew Cunningham, Mohsen Naghavi

**Affiliations:** I Universidade Federal de Minas Gerais, Departamento de Enfermagem Materno Infantil e Saúde Pública – Belo Horizonte (MG), Brasil.; II Universidade Federal de Minas Gerais, Escola de Enfermagem, Programa de Pós-Graduação em Enfermagem – Belo Horizonte (MG), Brasil.; III Universidade Federal Fluminense, Instituto de Matemática e Estatística, Departamento de Estatística – Niterói (RJ), Brasil.; IV Universidade Nova de Lisboa, Instituto de Higiene e Medicina Tropical – Lisboa, Portugal.; V University of Washington, Institute for Health Metrics and Evaluation – Seattle, WA, USA.; VI University of Washington, Institute for Health Metrics and Evaluation, Department of Global Health – Seattle, WA, USA.

**Keywords:** Rompimento de barragens, Mineração, Estudos ecológicos, Mortalidade, Causas externas, Diagnóstico da situação de saúde

## Abstract

**Objetivo:**

Descrever os padrões de mortalidade geral e por causas externas e a evolução temporal nos municípios da Bacia Hidrográfica do Rio Paraopeba previamente ao desastre socioambiental de Brumadinho e, adicionalmente, investigar a correlação entre a mortalidade e a privação socioeconômica nesses municípios.

**Métodos:**

Foram analisadas estimativas de mortalidade do Estudo Carga Global de Doenças referentes a 26 municípios de Minas Gerais. Calcularam-se taxas de mortalidade geral e por causas externas nos triênios (T) T1 (2000 a 2002), T2 (2009 a 2011) e T3 (2016 a 2018). O coeficiente de correlação de Pearson mediu associação entre as taxas de mortalidade e a privação socioeconômica, segundo Índice Brasileiro de Privação.

**Resultados:**

Houve declínio da mortalidade geral na Bacia Hidrográfica do Rio Paraopeba de 717,7/100.000 para 572,6/100.000 hab. e na maioria dos municípios entre T1-T3. A mortalidade por causas externas aumentou de 73,3/100.000 para 82,1/100.000 e foi mais elevada nesses municípios comparando-se com a média do Brasil e de Minas Gerais. As mortes por suicídio e violência interpessoal aumentaram de 29,6/100.000 para 43,2/100.000 na maioria dos 26 municípios. Os acidentes não intencionais reduziram-se no período, e as taxas por acidente de transporte aumentaram. Houve correlação positiva entre a privação socioeconômica e a variação percentual das taxas de mortalidade.

**Conclusão:**

Apesar da forte presença da atividade mineradora na região, isso não refletiu na melhoria do quadro sanitário, as causas externas aumentaram no período, associadas às desigualdades, o que deve ser considerado no planejamento para a recuperação das áreas do desastre.

## INTRODUÇÃO

O rompimento da Barragem I de contenção de rejeitos da Mina de Córrego do Feijão, no município de Brumadinho, Minas Gerais, em 2019, operada pela Companhia Vale S.A., provocou um dos mais graves desastres do mundo relacionados a barragens de mineração. Considerado o maior acidente de trabalho do Brasil, vitimou fatalmente 272 pessoas, entre as quais 250 funcionários diretos e terceirizados da Vale S.A.^1,2^. O desastre ocasionou o lançamento de, pelo menos, 12 milhões de metros cúbicos de rejeitos no solo e no Rio Paraopeba, que atingiram mais de 160 km de extensão^3^. Esses rejeitos resultaram em danos ambientais à vegetação e à fauna, bem como no lançamento de metais pesados na água, como manganês, alumínio, ferro, arsênio, restringindo seu uso e impedindo o abastecimento da água para a região metropolitana de Belo Horizonte^3,4^. Nesse curso, além de Brumadinho, foram considerados atingidos outros 25 municípios do estado^5^, que correspondem a uma população de cerca de 1,1 milhão de habitantes.

Os desastres, em geral, excedem a capacidade da comunidade ou sociedade afetada para enfrentar a situação com recursos próprios, o que pode ampliar os prejuízos e danos ao meio ambiente e à saúde, para além do local de ocorrência do evento em si^6^. São várias as consequências causadas, entre elas, prejuízos materiais e econômicos, além de doenças, agravos e óbitos também em momento posterior ao desastre^6^. Tais circunstâncias exigem decisões em caráter de urgência com o propósito de cessar ou diminuir os riscos ambientais que derivam da contaminação do solo e de cursos d’água e das exposições a eles e, consequentemente, mitigar os danos ao ecossistema e à saúde das populações, que podem surgir nos curto, médio e longo prazos^7^.

A literatura científica aponta para essas mudanças no perfil de morbimortalidade das populações afetadas pelos desastres, com aumento de prevalência das doenças crônicas e agudização das doenças que já estavam instaladas^7,8–15^, comprometimento da saúde mental, aumento do consumo de bebidas alcoólicas e outras drogas e da violência nas comunidades atingidas^16–20^. E a ocorrência das causas externas está relacionada ao processo de perda de familiares e entes queridos e à ruptura brusca nos processos sociais, econômicos e também identitários^21^. Para que seja possível dimensionar os impactos de um desastre, torna-se imperativo investigar as condições de saúde prévias, as vulnerabilidades e os contextos sociais e ambientais em que vivia a população afetada. Parte-se do pressuposto que as desigualdades sociais e a vulnerabilidade social resultam em piores indicadores em saúde na região, prévias ao desastre. Entende-se que conhecer o perfil de mortalidade e sua evolução temporal antes do desastre permitirá traçar um diagnóstico situacional e identificar tendências temporais cuja aplicabilidade — e relevância — consiste no estabelecimento de uma linha de base para futuras avaliações de impacto do desastre sobre a saúde das populações afetadas.

O presente artigo visa descrever os padrões de mortalidade geral e por causas externas e a evolução temporal nos municípios da Bacia Hídrica do Rio Paraopeba (BHRP) previamente ao desastre socioambiental de Brumadinho e, adicionalmente, investigar a correlação entre a mortalidade e a privação socioeconômica (Índice Brasileiro de Privação — IBP) nesses municípios.

## MÉTODOS

### Delineamento do estudo e unidade de análise

Trata-se de estudo epidemiológico do tipo ecológico, de caráter descritivo e analítico, que investigou a mortalidade geral e por causas externas em 26 municípios da BHRP, Minas Gerais (MG) (Material Suplementar – Figura A), entre 2000 e 2018, ou seja, em anos anteriores ao rompimento da barragem de contenção de rejeitos de mineração da Mina de Córrego do Feijão.

### Fonte dos dados

Foi utilizada a base de dados de óbitos por causas, por municípios, elaborada em 2021 por pesquisadores do Institute for Health Metrics and Evaluation (IHME), da Universidade de Washington, no âmbito do estudo Carga Global de Doenças (GBD), sob demanda da Rede GBD Brasil.

O estudo GBD utiliza dados nacionais coletados de sistemas de registro vital para estimar a mortalidade, e, no que concerne ao Brasil, a fonte de dados é o Sistema de Informações sobre Mortalidade (SIM), do Ministério da Saúde^22^. Visando à melhoria da qualidade da informação, o IHME aplica algoritmos para corrigir o sub-registro de óbitos e redistribuir óbitos classificados com códigos *garbage* (CG) entre óbitos atribuídos a causas específicas, consideradas causas básicas, conforme métodos previamente descritos em publicações científicas^23,24^. Óbitos classificados como *garbage* mascaram as verdadeiras causas básicas de óbito e correspondem a códigos da Classificação Estatística Internacional de Doenças (CID) que: não são suficientemente específicos; são causas intermediárias ou imediatas de morte; ou são causas impossíveis de morte^23^.

As estimativas de população elaboradas pelo Ministério da Saúde e disponibilizadas no *site* do Datasus^25^ foram utilizadas como denominador no cálculo das taxas de mortalidade.

### Indicadores

Foram calculadas as taxas de mortalidade geral e por causas externas, agregadas e separadamente, expressas por 100 mil habitantes, para cada um dos 26 municípios da Bacia do Rio Paraopeba e para o conjunto desses municípios. Para fins de comparação, também foram calculadas taxas de mortalidade para o conjunto de todos os municípios do estado de Minas Gerais e do Brasil. Nesse estudo, as causas externas compreenderam três grandes grupos de causas utilizados pelo GBD, no nível 2 (dois) de desagregação^23^: a) suicídio e violência interpessoal, b) acidentes não intencionais, c) acidentes de transporte. A fim de reduzir flutuações aleatórias, as taxas foram calculadas por triênios: T1 (2000/2001/2002), T2 (2009/2010/2011) e T3 (2016/2017/2018). O numerador compreendeu a média de óbitos e o denominador a população média de cada triênio. As taxas foram padronizadas para idade pelo método direto, utilizando-se a população padrão do estudo Carga Global de Doenças (“Global Burden of Disease” — GBD)^23^.

Foi empregado o IBP como medida de desigualdade social. O índice foi desenvolvido por pesquisadores do Centro de Integração de Dados e Conhecimentos para Saúde (Cidacs/Fiocruz Bahia) e da Universidade de Glasgow-Escócia. Trata-se de uma medida-síntese de privação socioeconômica de abrangência nacional calculada na escala de setores censitários, baseada em indicadores do censo demográfico de 2010 relativos à renda *per capita*, à alfabetização e ao acesso a saneamento básico^26^.

A soma dos indicadores foi realizada com base no cálculo do z-score: o “z”, para uma variável “x”, foi calculado usando a fórmula z=(x−μ)/sd, em que a média “μ” e o desvio-padrão “sd” para cada indicador do setor censitário foram ponderados de acordo com a população^26^. O valor final do z-score de cada setor censitário foi dado pela soma simples do z-score dos indicadores de renda, escolaridade e condições de domicílio^21^. O índice foi calculado em formato numérico também por municípios, em que a pontuação dos indicadores é somada em escore, possibilitando classificar cada município com um escore e agrupá-los em quintis de privação, em uma escala que varia da menor privação (-1,73) para a maior (+2,71). No caso dos 26 municípios da bacia, eles foram classificados entre -1,38 (2º quintil de privação) a -0,25 (4º quintil de privação) (Material Suplementar – Tabela A).

A base do IBP por municípios encontra-se disponível no *site* do Centro de Integração de Dados e Conhecimentos para Saúde (Cidacs/Fiocruz Bahia) para acesso publico^27^.

### Apresentação e análise dos dados

As taxas de mortalidade municipais foram apresentadas em tabela e mapas coropléticos. Percentuais de mudança das taxas de mortalidade entre os triênios foram apresentados nos gráficos do tipo mapa de calor (*heat map*), comparando-se as diferenças relativas entre o primeiro e o segundo triênios, o segundo e o terceiro, e entre o primeiro e o terceiro triênios. No mapa de calor, são apresentados os valores das variações percentuais das taxas entre os triênios, numa comparação dois a dois (T1-T2, T2-T3 e T1-T3). Esses valores são destacados segundo uma escala de cor que representa os maiores decréscimos (escala de azul) e os maiores incrementos das taxas (escala de vermelho).

Para a análise de correlação, considerou-se a relação entre a variação percentual das taxas de mortalidade do primeiro e terceiro triênios e o IBP. Utilizou-se o coeficiente de correlação de Pearson e foram consideradas correlações estatisticamente significativas as que apresentaram valor de p menor ou igual a 5%. Utilizou-se a classificação de Dancey e Reidy^28^ para definir a magnitude da correlação: valores menores que 0,30 são considerados fracos, entre 0,40 e 0,60 moderados e maiores que 0,70 fortes. A apresentação e a análise dos dados foi realizada no *software* estatístico R.

### Aspectos éticos

Este estudo integra projeto mais amplo aprovado pelo Comitê de Ética em Pesquisa da Universidade Federal de Minas Gerais sob o parecer de número 3.258.076. O estudo trabalhou com registros de dados secundários de base não nominal, que não permitem a identificação de indivíduos, em conformidade com o Decreto nº 7.724, 16 de maio de 2012^29^, e a Resolução nº 510, de 7 de abril de 2016^30^.

## RESULTADOS

As taxas médias de mortalidade geral para o conjunto dos 26 municípios da Bacia Hidrográfica do Rio Paraopeba declinaram de 717,7/100.000 hab. (T1) para 572,6/100.000 hab. (T3). O mesmo ocorreu para o Brasil, de 771,8/100.000 hab. (T1) para 622,6/100.000 hab. (T3), e para o estado de Minas Gerais (Tabela 1). Observa-se declínio da mortalidade geral no período em quase todos os municípios, com exceção de Fortuna de Minas, Papagaios, Paineiras e Igarapé, cuja tendência foi de acréscimo (Figura 1 e Material Suplementar – Tabela B).

**Tabela 1:** Taxas de mortalidade geral e por causas externas, padronizadas por idade, por 100 mil habitantes, nos triênios T1 (2000/2001/2002), T2 (2009/2010/2011) e T3 (2016/2017/2018), Brasil, Minas Gerais e conjunto dos municípios da Bacia Hidrográfica do Rio Paraopeba.

Local	Mortalidade geral			Causas externas			Suicídio e violência interpessoal			Acidentes não intencionais			Acidentes de transporte		
	T1	T2	T3	T1	T2	T3	T1	T2	T3	T1	T2	T3	T1	T2	T3
Brasil	771,8	669,6	622,6	84,9	79,3	75,1	37,6	34,8	36,8	21,8	20,2	18,5	25,5	24,3	19,7
Minas Gerais	698,8	606,0	553,1	62,2	70,9	64,4	22,4	29,9	29,2	18,7	17,4	16,2	21,1	23,6	18,9
Bacia Rio Paraopeba*	717,7	625,2	572,6	73,3	91,2	82,1	29,6	45,5	43,2	20,1	18,1	17,5	23,7	27,7	21,4

* Corresponde ao conjunto dos 26 municípios da Bacia Hidrográfica do Rio Paraopeba.

No que se refere à mortalidade pelo conjunto das causas externas, ocorreu o inverso, com aumento das taxas de mortalidade na Bacia do Paraopeba, de 73,3/100.000 hab. (T1) para 91,2/100.000 hab. (T2) e 82,1/100.000 hab. (T3), enquanto as taxas do Brasil declinaram de 84,9/100.000 hab. (T1) para 75,1/100.000 hab. (T3); e em Minas Gerais se mantiveram estáveis de 62,2/100.000 hab. (T1) para 64,4/ 100.000 hab. (T3) (Tabela 1). A Figura 2 mostra taxas mais elevadas nos municípios da Bacia do Paraopeba nos dois últimos triênios em relação ao primeiro. Entre os municípios da Bacia do Paraopeba, ocorreu aumento nas taxas ao longo dos três triênios na maioria dos municípios e alguns com taxas muito elevadas, como: Papagaios, Fortuna de Minas e Pompéu no T3 (135,5 por 100 mil habitantes, 117,1 por 100 mil habitantes e 114,6 por 100 mil habitantes, nessa ordem) (Material Suplementar – Tabela B).

**Figura 1:** Taxas municipais de mortalidade geral, padronizadas por idade, por 100 mil habitantes, nos triênios T1 (2000/2001/2002), T2 (2009/2010/2011) e T3 (2016/2017/2018), Bacia Hidrográfica do Rio Paraopeba, Minas Gerais.

**Figura 2:** Taxas municipais de mortalidade pelo conjunto das causas externas, padronizadas por idade, nos triênios T1 (2000/2001/2002), T2 (2009/2010/2011) e T3 (2016/2017/2018), Bacia Hidrográfica do Rio Paraopeba, Minas Gerais.

A Figura 3 mostra os valores das variações percentuais das taxas de mortalidade entre os triênios. Quanto ao conjunto das causas externas, observou-se, entre T1-T2, aumento de +24,4% na Bacia do Paraopeba, e, ao contrário, redução de -6,6% no Brasil, e aumento de +13,9% em MG. Entre T1-T3, houve incremento de +12,0% na Bacia do Paraopeba e +3,5% em MG e redução de -11,6% no Brasil. Analisando-se os municípios separadamente, ocorreu aumento das taxas entre os triênios, sobretudo de T1-T2 em quase todos os municípios, exceto Caetanópolis e São Gonçalo do Abaeté. Os maiores acréscimos ocorreram em Paineiras (+120,6%), Papagaios (+75,1%) e Igarapé (+57,3%). No T1-T3, houve leve redução em uma quantidade maior de municípios (Figura 3).

**Figura 3:** Variação percentual das taxas de mortalidade geral, por causas externas totais e desagregadas, entre os triênios T1 (2000/2001/2002), T2 (2009/2010/2011) e T3 (2016/2017/2018), no Brasil, em Minas Gerais e nos municípios da Bacia Hidrográfica do Rio Paraopeba.

Nota: R: coeficiente de correlação de Pearson; p: valor de p do teste de correlação; IBP: Índice Brasileiro de Privação.

Avaliando as causas externas desagregadamente, com recorte para o conjunto dos 26 municípios da Bacia do Paraopeba, o suicídio e a violência interpessoal responderam pela maior carga de morte, seguidos dos acidentes de transporte e, por fim, dos acidentes não intencionais (Tabela 1). No terceiro triênio, a taxa de mortalidade por suicídio e violência interpessoal foi duas vezes o valor da taxa de mortalidade por acidente de transporte (43,2 *vs* 21,4 por 100 mil habitantes) e 2,5 vezes o valor da taxa média de mortalidade por acidentes não intencionais (43,2 *vs* 17,5 por 100 mil habitantes) (Tabela 1). No que se refere à mortalidade por suicídio e violência interpessoal, as taxas da Bacia do Paraopeba foram mais altas que as verificadas para Minas e para o Brasil no segundo e terceiro triênios (Tabela 1). Na bacia do Paraopeba, observou-se aumento de mais de 50% do primeiro para o segundo triênio e de 46,2% do primeiro para o terceiro triênio, enquanto, no Brasil, ocorreu estabilidade na variação percentual entre T1-T2 e T1-T3 e, em Minas, houve incremento de mais de 30% do primeiro para o segundo triênio (Figura 3). Ao longo do período analisado, houve aumento expressivo das taxas na maioria dos municípios (Material Suplementar – Figura A, Tabela B; Figura 3).

As taxas de mortalidade por acidentes não intencionais foram semelhantes para Brasil, Minas Gerais e Bacia do Paraopeba (Tabela 1). A Figura B do Material Suplementar mostra um escurecimento do mapa de T1 para T2 e um clareamento de T2 para T3, indicando, ao longo dos três períodos, acréscimo das taxas seguido de decréscimo. As taxas reduziram-se entre T1 e T3 na maioria dos municípios (n=14) e ficaram estáveis em outros oito municípios (Material Suplementar – Tabela B; Figura 3).

Quanto à mortalidade por acidentes de transporte, as taxas mais altas em T3 ocorreram na Bacia do Paraopeba (21,4 por 100 mil habitantes), em comparação com as taxas médias do Brasil (19,7 por 100 mil habitantes) e de Minas Gerais (18,9 por 100 mil habitantes) (Tabela 1). A Figura C do Material Suplementar mostra que as taxas aumentaram nos municípios especialmente entre T1-T2, reduzindo-se levemente no último triênio. Comparando-se T1-T3, ocorreu redução nas taxas por acidente de transporte em 12 municípios e incremento acima de 10% em 8 municípios (Figura 3).

Na análise de correlação entre o IBP e as causas de mortalidade, identificou-se correlação positiva de magnitude moderada e estatisticamente significativa entre: a variação percentual das taxas de mortalidade por acidentes não intencionais e o IBP (R=0,49; p=0,012); e a variação percentual das taxas de mortalidade por acidentes de transporte e o IBP (R=0,42; p=0,035) (Figura 4).

**Figura 4:** Gráfico de dispersão e análise de correlação entre a variação percentual das taxas de mortalidade geral e por causas externas nos triênios T1 (2000/2001/2002) e T3 (2016/2017/2018) e o Índice Brasileiro de Privação de cada município da Bacia Hidrográfica do Rio Paraopeba, Minas Gerais.

## DISCUSSÃO

O estudo identificou elevadas taxas de mortalidade geral e por causas externas nos municípios da Bacia do Rio Paraopeba, mais elevadas que a média do Brasil e de Minas Gerais no período pré-desastre ambiental da Empresa Vale S.A. em Brumadinho. Entre as causas externas, as mortes por suicídio e violência interpessoal foram as mais elevadas e aumentaram ao longo dos triênios nos 26 municípios. Os acidentes não intencionais reduziram-se no período, e as taxas por acidente de transporte aumentaram. Estas últimas tenderam a aumentar conforme o aumento da privação socioeconômica, medido pelo IBP.

O desastre ocorrido em 2019 no município de Brumadinho explicita a negligência com as questões ambientais, sociais, de saúde e bem-estar da população afetada e não condiz com o comprometimento do país com pactos globais, como o Acordo de Sendai e a Agenda 2030 de desenvolvimento sustentável. O Marco de Sendai para a Redução de Riscos de Desastres tem como objetivo alcançar, até 2030, a redução substancial de riscos de desastres e de perdas de vidas, com ações dedicadas e específicas de boa governança^31^. A meta 3.9 dos Objetivos de Desenvolvimento Sustentável (ODS) prevê reduzir substancialmente o número de mortes e doenças por produtos químicos perigosos, contaminação e poluição do ar, da água e do solo^32^.

Um desastre traz inúmeras consequências e repercussões para as pessoas e comunidades atingidas. Ao mesmo tempo, e paradoxalmente, ele também representa a possibilidade de revelar as condições históricas e sociais que se encontram por detrás de sua origem e podem servir para fomentar discussões para a transformação destas^33^. Antes desse desastre, as comunidades já vivenciavam vulnerabilidades e ausências de estruturas e políticas públicas; as elevadas taxas de mortalidade por violências explicitadas neste estudo sugerem esse cenário. E, para compreender o perfil de mortalidade de uma população, deve-se buscar a explicação nos processos sociais^34^.

Tomando por base o IBP, os municípios com maior privação socioeconômica apresentaram taxas mais altas de mortalidade geral e por causas externas no período pré-desastre. Os municípios mais violentos da bacia podem ser separados em dois grupos: os mais próximos da região metropolitana de Belo Horizonte (Betim, Juatuba, Esmeraldas e São Joaquim de Bicas) e os munícipios que se localizam no baixo Paraopeba (Papagaios, Pompéu e Paineiras). Esse segundo grupo tem como característica forte relação com a agropecuária e a mineração^3,35^.

A região da Bacia do Paraopeba teve aumento, no período prévio ao desastre, das mortes por causas externas, especialmente por suicídio, violência interpessoal e acidente de transporte. Destaca-se o aumento da violência entre o primeiro e o terceiro triênios, muito superior ao aumento das taxas de Minas e do Brasil. Esses achados podem refletir a contradição já identificada em outros estudos sobre desenvolvimento econômico e mineração. Se, por um lado, os grandes projetos de mineração geraram crescimento econômico, por outro lado, não geraram maior bem-estar da população em geral^36^. Destaca-se que a atividade mineradora consiste em uma das principais atividades econômicas do país, responsável por cerca de 4,08% do produto interno bruto (PIB) em 2018. Contudo, junto com o seu impacto econômico, apresenta expressivas externalidades socioambientais, degradação do meio ambiente e desastres socioambientais^37^. Estudo realizado no Pará, em seis municípios sede de grandes mineradoras, identificou que, apesar do crescimento econômico significativo puxado pela indústria extrativista, não houve alteração na forma estrutural das condições de pobreza dos municípios. Ao contrário, ocorreram retrocessos do ponto de vista das desigualdades sociais, com piora do índice de Gini entre 1990 e 2010. Ou seja, a mineração ampliou a concentração de renda e aumentou as desigualdades sociais^36^. Outro estudo destaca ainda o baixo investimento na capacidade regulatória do estado sobre a atividade mineradora, sendo o estado incapaz de fiscalizar e regular de forma adequada essa importante atividade econômica^37^. Assim, esses estudos podem ajudar a compreender os resultados na região da Bacia do Paraopeba. Apesar da forte presença da atividade mineradora nas últimas décadas na região e do aumento do ritmo de extração, não houve melhora no seu quadro sanitário, persistindo problemas estruturais, desigualdades sociais, vulnerabilidades locais que precisam ser superados.

Lesões e morte por causas externas resultam em ônus social, sanitário e econômico para indivíduos, famílias, sociedade e governo e consistem em importante problema de saúde pública no Brasil^38^. O Brasil destaca-se por ser um dos mais violentos do mundo: o segundo com mais mortes por armas de fogo^39^ e um dos cinco países com taxas mais elevadas de mortes no trânsito. Segundo Minayo^40^, a violência estrutural está institucionalizada na sociedade, sistemas econômicos, culturais, políticos e família, influencia profundamente as praúticas de socializacão e resulta em sofrimento e morte.

Fatores socioeconômicos estão diretamente relacionados à violência; esta resulta também das desigualdades sociais e afeta principalmente locais de miséria e pobreza^41^. Portanto, para superar esse quadro, é preciso fortalecer a vigilância em saúde, fomentar a articulação intersetorial, interdisciplinar e multiprofissional e a organização da sociedade civil e comunitária^42^, promover pesquisas para identificação de políticas e estratégias de prevenção da violência, bem como apoiar o monitoramento e a avaliação da efetividade das acões^40,43^.

O estudo apresenta fortalezas, como o uso dos dados de óbito do GBD, ajustados para subregistro e com redistribuição de códigos *garbage*, o que significa um avanço no campo da melhoria da informação de mortalidade. Contudo, trata-se de estudo ecológico, que tem limites na sua capacidade de análise e atribuição de causalidade, agrega-se ainda o fato de serem dados referentes a municípios pequenos, as taxas podem apresentar flutuações decorrentes dos pequenos números no numerador, as quais se buscou minimizar pela agregação em triênios. As causas externas foram desagregadas no nível 2 do estudo GBD, que são mais agregados e incluem a violência interpessoal e o suicídio, embora a maior fração das taxas de mortes sejam por agressões, dados não mostrados. Outra limitação diz respeito ao IBP, cujos indicadores foram calculados com base em variáveis de 2010 por não existirem dados atualizados do censo demográfico. Há que se considerar, portanto, a implicação da defasagem desses dados sobre a classificação da privação nos municípios, podendo resultar em viés no diagnóstico de vulnerabilidade. Por fim, importante mencionar que o coeficiente de correlação de Pearson quantifica somente o grau da relação linear entre duas variáveis e que altas correlações não implicam relação de causa e efeito.

Como conclusão, apesar da forte presença da atividade mineradora na região da bacia do Paraopeba, persistem as situações de desigualdades sociais, tampouco houve melhoria do quadro sanitário no período anterior ao desastre. As causas externas aumentaram e estão associadas às desigualdades sociais, que são acentuadas na região. Esses dados devem ser considerados nas medidas de prevenção a serem implantadas e podem orientar o direcionamento dos recursos que serão investidos na região, como compensação aos danos ambientais produzidos. Esses resultados poderão instrumentalizar as partes interessadas e apoiar ações destinadas aos grupos populacionais afetados e mais vulnerabilizados.
